# Grouping and Segregation of Sensory Events by Actions in Temporal Audio-Visual Recalibration

**DOI:** 10.3389/fnint.2016.00044

**Published:** 2017-01-19

**Authors:** Nara Ikumi, Salvador Soto-Faraco

**Affiliations:** ^1^Multisensory Research Group, Center for Brain and Cognition, Universitat Pompeu FabraBarcelona, Spain; ^2^Institució Catalana de Recerca i Estudis AvançatsBarcelona, Spain

**Keywords:** multisensory, cross-modal, temporal recalibration, audio-visual, motor synchronization, action, attention

## Abstract

Perception in multi-sensory environments involves both grouping and segregation of events across sensory modalities. Temporal coincidence between events is considered a strong cue to resolve multisensory perception. However, differences in physical transmission and neural processing times amongst modalities complicate this picture. This is illustrated by cross-modal recalibration, whereby adaptation to audio-visual asynchrony produces shifts in perceived simultaneity. Here, we examined whether voluntary actions might serve as a temporal anchor to cross-modal recalibration in time. Participants were tested on an audio-visual simultaneity judgment task after an adaptation phase where they had to synchronize voluntary actions with audio-visual pairs presented at a fixed asynchrony (vision leading or vision lagging). Our analysis focused on the magnitude of cross-modal recalibration to the adapted audio-visual asynchrony as a function of the nature of the actions during adaptation, putatively fostering cross-modal grouping or, segregation. We found larger temporal adjustments when actions promoted grouping than segregation of sensory events. However, a control experiment suggested that additional factors, such as attention to planning/execution of actions, could have an impact on recalibration effects. Contrary to the view that cross-modal temporal organization is mainly driven by external factors related to the stimulus or environment, our findings add supporting evidence for the idea that perceptual adjustments strongly depend on the observer's inner states induced by motor and cognitive demands.

## Introduction

Perception requires actively processing multiple sources of information arriving to the brain from various sensory modalities. A fundamental issue in multisensory perception is how the system combines sensory signals to achieve a coherent representation of the environment. Temporal coincidence amongst inputs in different modalities is generally claimed to play a paramount role in guiding the organization of events into multisensory objects (Meredith et al., 1987). However, at the time information is available via different sensory modalities, the original temporal relations of the events at source are not preserved. This is due to differences in transmission velocities between different sorts of physical energies as well as different transduction and neural processing times between sensory pathways (e.g., King, 2005). Moreover, it is well known that the subjective experience of cross-modal synchrony is influenced both by external and cognitive factors other than time difference itself, such as spatial proximity (Zampini et al., 2005), recent prior experience of temporal order across modalities (Fujisaki et al., 2004; Vroomen et al., 2004; Van der Burg et al., 2013) and, training with feedback (Powers et al., 2009), amongst others. Consequently, physical simultaneity and perceptual simultaneity (the point of subjective simultaneity, or PSS) are far from being perfectly correlated (King and Palmer, 1985). The variety of factors shaping synchrony perception motivates the need for internal adjustment processes to flexibly calibrate the timing of events between sensory modalities. This flexibility in inter-sensory adjustment processes might help adapt to different conditions of the environment, at the expense of handling a certain degree of uncertainty in the temporal domain. An important strand of evidence for flexible temporal adjustments is the phenomenon of temporal recalibration, showing that exposure to a fixed temporal asynchrony between cross-modal pairs (adaptation phase) recalibrates the point of subjective synchrony for subsequent cross-modal pairs, toward the adapted asynchrony direction (Fujisaki et al., 2004; Vroomen et al., 2004). For example, if participants adapt to flashes leading sounds by 200 ms repeatedly presented during a minute, synchrony perception shifts, such that events in which the flash is presented slightly before the sound (same adapted direction) are more likely to be perceived simultaneous than after participants have been adapted to the reverse modality order (sounds leading flashes by 200 ms).

Despite the phenomenon has been well described, the mechanism underlying the process of cross-modal temporal recalibration is still unclear. One outstanding question is whether the re-alignment between sensory modalities is constrained to the modalities involved in the recalibration process. A modality-dependent account is in line with the co-existence of multiple clocks, in which the perceptual latencies of one of the sensory signals is speeded up or slowed down relative to the other (Navarra et al., 2007, 2009; Harrar and Harris, 2008). Consistent with this latency shift account, Di Luca et al. (2009) found that the more reliable modality in the immediate context was the one that would be taken as a reference to recalibrate the other modality/ies. The alternative, modality independent account, owes to a common supra-modal process relying on a centralized clock (Hanson et al., 2008). In line with the supra-modal framework, Yarrow et al. (2011) proposed a model relating modulations in time perception to a decisional level of processing, in particular, a shift in the criterion used to judge synchrony. Alternatively, other theoretical accounts are based on the modulation of prior likelihood distributions about modality order, learnt from contingencies in the environment (Yamamoto et al., 2012), and/or the adaptation of lag-sensitive neurons (Roach et al., 2011).

In most cases, prior studies to back up these accounts have only considered the interplay between sensory inputs (in different modalities). However, in real life situations, inputs from different modalities often arise in the context of sensori-motor interactions. Hence, in the present study we decided to consider the role of actions in the temporal recalibration between sensory modalities. The proposal that the motor system might play a role in sensory processing is based on the idea that anticipatory or cross-modal predictive processes are key during temporal adjustment (van Atteveldt et al., 2014). Following this account, the temporal recalibration process would engage general predictive systems grounded on the motor domain. This is not a new idea. An interesting framework along these lines proposes that perceptual timing could be in fact calibrated based on motor interactions with the environment (Stetson et al., 2006; Eagleman, 2008). Instead of direct temporal adjustments between sensory systems, like the aforementioned accounts propose (Navarra et al., 2009, 2007; Harrar and Harris, 2008; Di Luca et al., 2009), this approach suggests that actions might provide a reference for temporal adjustment of sensory modalities; for example re-aligning the timings of the sound and feel of our footsteps to walking movements, or the visual, tactile and auditory consequences of an action that produces sensory information (e.g., playing the piano, knocking a door, hand clapping). Consistent with this idea, several prior studies have shown that, similar to cross-modal temporal recalibration, motor-sensory temporal recalibration occurs when a delayed sensory event is systematically presented following participants' actions (Stetson et al., 2006). In Stetson's study, the adaptation phase consisted of a flash presented at a fixed interval, right after participant's button presses. Subsequently, when a flash was presented right at the time of the button press in a test phase, participants would often perceive that the flash occurred prior to their action. Eagleman (2008) proposed that recalibration might reflect prior expectations about motor-sensory temporal organization. In agreement with this interpretation, a recent study has demonstrated that specific learned associations between actions and sensory outcomes during adaptation can modulate temporal adjustments based on participants' expectations (Desantis et al., 2016). Moreover, given that magnitudes of motor-sensory recalibration are usually much larger than those observed with sensory-sensory recalibration, Eagleman (2008) claimed that active interaction with the environment might be the most efficient way to calibrate the mental time of events. In fact, numerous studies investigating the neural substrates of time perception have highlighted the recruitment of motor circuits during the execution of perceptual tasks (Coull and Nobre, 2008). Other studies go further and propose that actually, the predictive core of the sensory-motor system might be shared both, by action control and the perception of sensory events (Engel and Fries, 2010 for a review; Schubotz, 2007).

Based on this potential role of actions in perception, perhaps the importance that has traditionally been given to the experience of relative timing (exact temporal order between sensory events) in multisensory perception should be reconsidered (Kopinska and Harris, 2004; for a review see Holcombe, 2013). That is, the question of to which degree one modality adapts to another may not be so relevant after all. Instead, general anchoring processes ground on the motor system might play a fundamental role determining grouping and segregation of sensory inputs (in line with Holcombe, 2013). While most multisensory studies involve situations that comprise the concurrent coordination of actions in time, only a few studies have directly explored the influence of actions on cross-modal temporal recalibration. In a series of experiments, Parsons et al. (2013) examined whether motor actions could influence audio-visual temporal adjustments. They asked participants to report the simultaneity of audio-visual events presented at two different distances from the observer (near and far condition) during a passive (only audio-visual) and an active (action triggers audio-visual events) condition. In the passive condition the sound was always presented at a fixed delay after the start of a trial, whereas in the active condition it was presented after a button press, and the flash was presented at variable intervals before or after the sound. The results indicated that the perceived time of the sound shifted in time only in the active condition. The authors interpreted the isolated perceptual re-alignment of the sound as an evidence against temporal adjustments resulting from perceptual interval compressions (or intentional binding. i.e., see Haggard et al., 2002) between actions and subsequent sensory events (in line with Cravo et al., 2011). Contrary to the prediction of the intentional binding account, Parsons et al. result suggests that temporal adjustments only affected events (sounds in this case) systematically linked to actions (by way of a reliable temporal interval). In addition, when far and near conditions were taken into consideration, the authors found that participants could compensate the temporal delays due to different transmission speeds between light and sound only in the active condition. This finding suggests the importance of active interactions in order to calibrate information arriving at different senses. Completing these series of experiments, Parsons et al. demonstrated that temporal re-alignment of information could be transferred from sensory-motor to sensory-sensory event pairs. This last result suggests that temporal alignment through motor interactions might be a possible mechanism for the adjustment between sensory events, even during passive exposure. Please note that, as in Parsons et al. (2013) we use the term “action” to refer to a volitional component as well as a purely proprioceptive signal. Following Kawabe et al. (2013) we consider that both components could be acting as temporal anchors, but the particular dissociation between them in the temporal re-alignment of the sensory events is out of the scope of the present study.

In the present study, we investigate whether actions that putatively induce audio-visual grouping or segregation, can determine the strength of cross-modal temporal recalibration. We speculate that motor task demands might not only produce temporal anchoring *per se*, but guide participant's attention. It is known that the focus of attention during adaptation can induce important modulations in temporal recalibration (Heron et al., 2010; Ikumi and Soto-Faraco, 2014). In particular, Heron et al. (2010) demonstrated that the magnitude of recalibration is larger when the observer focuses attention to the temporal order of flash-tone pairs, compared to attending to only one of the modalities, to the fixation cross or to the inter-trial intervals. Ikumi and Soto-Faraco (2014) showed that the focus of attention can modulate the direction of cross-modal recalibration. We argue that the execution of actions might lead attention to shift to the particular events occurring within the time of that action, and subsequently shape their temporal relationship. The purpose of the present study was to examine whether the magnitude of recalibration between simple flash-tone pairs can be modulated as a function of a tapping synchronization task^1^ targeting the same sensory events used for adaptation. The synchronization of actions was carried out during the adaptation phase, putatively inducing perceptual grouping vs. segregation of the co-occurring sensory events. Hence, by hypothesis we expect (stronger) recalibration (measured as a PSS shift between different directions of adaptation) when the nature of the motor task promotes grouping and absence (or weaker) recalibration when the motor task promotes segregation of audio-visual events.

## Materials and methods

### Ethics statement

All procedures had been previously approved by the local ethical board (CEIC Parc de Mar). Informed consent from all participants was obtained in written, prior to the start of the experiment.

### Participants

Sample group size for each of the three experimental conditions (grouping, segregation, control) was set a priori to *N* = 16. Thus, 48 participants were analyzed (ages between 18 and 35 years old). Fourteen additional participants (4, 6, and 4, respectively, in the grouping, segregation and control conditions) were excluded from the analysis and replaced to obtain 16 participants per group, because of low performance levels in the simultaneity judgment or the synchronization task (see the exclusion criteria in the data analysis section). All participants were paid volunteers who were naive about the purpose of the experiments, plus two of the experimenters (N.I. and O.V.). All had normal or corrected-to-normal vision and hearing.

### Apparatus

The experiments were run on a PC using Psychotoolbox libraries on Matlab R2010b. The participant sat at the distance of 60 cm from the monitor (“PHILLIPS109B,” 85 Hz, 800 × 600 pixels), in a quiet dark room. The auditory stimuli were presented through headphones (Sennheiser PC 161). Accurate timing and synchronization of auditory and visual stimuli was achieved using a Blackbox Toolkit (Accuracy of <1 ms; Cambridge Research Systems). Flexion and extension finger movements were captured by an optical sensor ROS-W Remote Optical Sensors (Speed Range of 1-250.000 RPM, Monarch Instrument). The sensor was mounted in a box that contained cushioned hand and arm support, for the participant's comfort during the experiment.

### Stimuli

The visual stimulus was a ring (outer and inner diameter, 4.2° and 2.1°, respectively) that flashed for one monitor frame (11.8 ms) at the center of a black square pedestal (9.7°) on light gray background (58.4 cd/m^2^). A fixation cross was presented at the square's center. The auditory stimulus was a tone (1.8 kHz, ~60 [A]dB SPL) lasting for 10 ms with a 2.5 ms raised-cosine ramp at the onset and offset.

### Procedure

Three groups of participants were tested in a temporal recalibration paradigm (adapted from Fujisaki et al., 2004). Typically this paradigm measures how audio-visual simultaneity perception in test trials changes as a function of an exposure period (adaptation phase) where pairs of audio-visual events were presented at a constant asynchrony. In this study, the adaptation included the performance of distinct action types (grouping, segregation and control condition). The actions were intended to promote either grouping or segregation of events in time. In particular, the action task in the grouping condition encouraged to group the action with a pair of events and in the segregation condition, the task encouraged to individuate actions to each one of the two single events. The actions were either synchronized to specific sensory modalities (to flashes or/and tones) or to specific time points in the absence of any event (see below for the detailed description of each condition).

**Figure 1:**
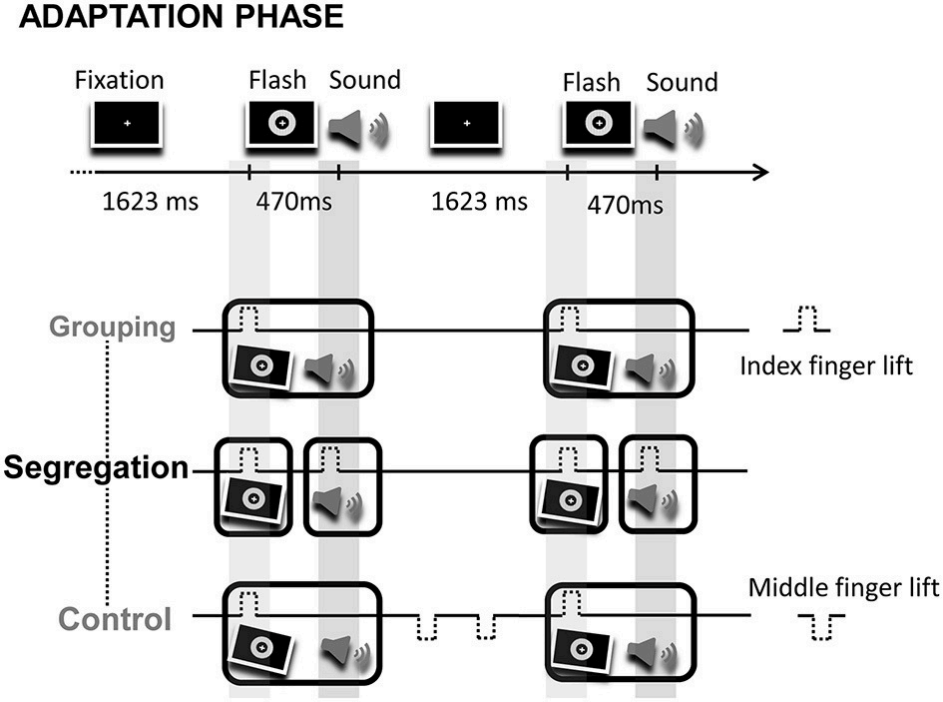
**Illustration of a fragment of an adaptation phase**. On the top of the image the screen display is depicted, consisting on the presentation of flash-sound pairs at a fixed rhythm. Below, the corresponding motor task for the grouping, segregation and control action type groups. The motor task consisted in the synchronization of finger lifts (index or middle finger) to flashes, sounds and/or to blank intervals. The tilted flashes and sounds surrounded by boxes represent the predicted temporal adjustment between flashes and sounds, fostering perceptual grouping (in the grouping and control groups) or segregation (in the segregation group) after adaptation.

During the adaptation phase, participants were exposed during 1 min to audio-visual pairs where flashes preceded tones (VA), or to audio-visual pairs where flashes lagged tones (AV) by 470 ms. Each participant ran the two adaptation modality orders, but in separate days (modality order counterbalanced between participants). The interval between consecutive flash-tone (or tone-flash) event pairs remained constant throughout the adaptation phase (ISI = 1623 and 1630 ms, respectively). Unlike the classical recalibration paradigm where participants simply detected deviant events or changes on the temporal structure of events during the adaptation period, here, participants were instructed to actively execute actions (finger lifts). Specifically, in the grouping condition, participants were asked to synchronize finger lifts to the leading or lagging event (1 action). Note that in this case, because of the fixed cross-modal lag, the action synchronizes to the sequence of the two sensory events (in either order; counterbalanced between participants). Alternatively, in the segregation condition, participants were asked to synchronize finger lifts to each event in the pair (2 actions). Finally, in the control condition participants performed 3 actions. The motor task of the control condition was intended to generate similar effects to the action in the grouping condition, but inducing additional cognitive load in order to control for possible effects arising from having to execute different number of actions on the grouping and segregation tasks. The first action required synchronizing a finger lift to the leading event (a flash or a tone), just like in the grouping condition. Two extra finger lifts were also carried out once the stimuli pair had disappeared, in the interval between audio-visual pairs. Figure 1 shows a fragment of the adaptation phase and the motor tasks with the corresponding predicted perceptual adjustments for each action type condition after adaptation.

Following the adaptation, participants alternated 9 s of re-adaptation trials (top up) with test trials, in which a simultaneity judgment (SJ) task was performed. The re-adaptation trials appear to be necessary to maintain the recalibration after-effects during the test phase (Machulla et al., 2012). The color of the fixation cross appearing at the center of the screen informed participants about whether the current pair was part of the (re)adaptation period (in which case they had to synchronize finger lift actions) or the simultaneity judgment (SJ) of the test trial. As long as the fixation cross in the center of the screen was white, participants were instructed to perform the synchronization task. Whenever the fixation cross changed to red, they stopped synchronizing their actions with the events, and prepare for the SJ. The fixation cross remained red for 1700 ms before the presentation of the test trial, a flash preceded or followed by a tone displayed at one of the following stimulus onset asynchronies (SOAs); −353 ms; ±235 ms; ±118 ms; ±59 ms; 0; +412 ms. A negative value denotes the tone preceded the flash, and a positive value denotes the reverse modality order. Half a second after the test trial presentation, a response screen was displayed in which two keys (“z” and “x”) were randomly assigned to simultaneity or non-simultaneity responses. Here, participants were asked to judge the simultaneity of the presented flash-tone pair. They were instructed to wait until the response screen was presented and informed that accuracy was preferred from speeded key presses.

Each participant ran two adaptation conditions (VA or AV) in consecutive days (order of adaptation conditions was counterbalanced across participants and action type). Each session was divided in three blocks of 10 min each, separated by a few minutes' breaks. A block consisted of a one-minute adaptation phase, followed by a test phase, where each SOA was presented 4 times, chosen equiprobably and at random. Overall, there were 12 repetitions per SOA and adaptation condition (216 test trials).

Participants were familiarized to the SJ task during <5 min prior to each experimental session (no feedback about the performance was given). Before the first experimental session each participant ran a short Pretest block, which consisted of a SJ task with the 9 SOAs tested during the experimental session. The psychophysical curves of the Pretest block were analyzed on the spot for each participant. If the bootstrap (Bca method from Efron, 1987) procedure failed to estimate the confidence intervals (CI) of the desired estimated parameters (for further details see the data analysis section) for a given participant, the participant was not selected for the experimental session. In these cases, we considered that parameter estimation was not reliable in that participant due to noise or the need of larger SOAs. This particular limitation about the size and number of SOAs might have skewed participants in this study with narrow temporal windows, which can result in smaller magnitudes of recalibration (Van der Burg et al., 2013). Subsequently, participants were trained to perform the motor task until their synchronization performance reached the 70%. Bear in mind the motor task consisted in synchronizing finger lifts with sensory events or specific time points. Feedback from the motor task was provided to the participants. Synchronization training could last from 10 to 30 min. Participants who were not able to synchronize after several training blocks were discarded.

To correctly measure the exact timing of the finger lifts, we employed an infrared light emitting diode (LED) as a movement sensor. Each action consisted of an extension (finger lift crossing the infrared beam) and a flexion (finger back to the resting position) of the finger. Finger lifts on the air were used because they do not involve tactile information. In this way, we intended to avoid adding another source of sensory interaction that could have interfered the perceptual recalibration between the sound and the flash (Roseboom et al., 2009). Feedback about the synchronization performance was given to the participants during the breaks between blocks. This helped maintaining the motivation throughout the experiment. However, no feedback about the SJ task was given during the experiment.

In the study we employed an audio-visual lag of 470 ms due to fingers' synchronization constraints. Despite the common lags used in temporal recalibration studies range from 100 to 250 ms, recalibration has been reported at lags spanning up to 700 ms when vision leads audition (Navarra et al., 2013). Even so, to ensure recalibration can be obtained with this relatively long lag, a pilot study with 6 participants was run (described in the Supplementary Materials).

### Data analyses

Participants' responses on the SJ task in each condition were assessed as the proportion of “simultaneous” responses at each SOA. Then, in each condition and participant, the data were fitted using the maximum likelihood estimation method into two cumulative Gaussian functions, each used to model each side of the curve (positive and negative SOAs). This type of fit is used to capture possible asymmetries between the two sides of the psychophysical function (for further details see Yarrow et al., 2011). The midpoints of each of these two Gaussian functions reflect the boundaries assumed to be used by an observer when having to judge simultaneity. The model used in our analysis employed three free parameters^2^: two boundaries [one for audition leading (A50V) and one for vision leading (V50A)] and one standard deviation (SD) of the cumulative Gaussian curves for both boundaries, which reflects the temporal window of simultaneity. The maximal perception of subjective simultaneity (PSS) was estimated as the midpoint between these two boundaries.

We were interested in comparing the magnitude of recalibration after-effects between groups (grouping, segregation and control), that differed on the motor task instructions during adaptation. The recalibration magnitude (PSS shift) was obtained by subtracting PSS values following the adaptation to VA pairs, from PSS values after adaptation to AV pairs. Moreover, we examined the SD values after the adaptation conditions between groups of interest (grouping, segregation and control). Because our hypothesis does not consider separate predictions for the A50V and V50A boundaries, the separate analysis is not included in the main text (see the results and corresponding analysis in the Supplementary Materials). Finally we also report the response time performance of the motor synchronization task in AV and VA adaptation conditions. The time of the participants' finger lift (as it crossed the infrared light beam) was measured relative to the target event. We extracted the errors of synchronization by measuring the proportion of finger lifts outside a ±250 ms window relative to the onset of the event (synchronization intervals adapted from finger tap studies using asynchrony ranges of −250 ms to +100 ms i.e., Sugano et al., 2012). In the control condition, where some actions were required to be executed in the period between events, we considered correct synchronization when actions were performed during a 500 ms window after stimulus offset (between +10 and +510 ms relative to the event; and the second finger lift performed between +210 and +700 ms relative to the event).

Data from 8 participants were excluded from the analysis due to failure of the fitting curve criteria (same criteria applied for the SJ task in the Pretest, see procedure section) during the experimental session in both adapted sessions (4 from the grouping, 3 from the segregation and 1 from the control group). Other 6 participants were excluded because their synchronization performance was below 50% synchronization in at least one of the sessions (3 from the segregation group and 3 from the control condition). Bad sensorimotor synchronization performances cannot ensure participants' actions were promoting grouping or segregation of sensory events in time. The exclusion of these participants could have skewed participants with better sensorimotor coordination in our study.

The statistical analyses were conducted with the IBM SPSS Statistics 19 software package. Effect sizes were calculated following (Lakens, 2013), using the spreadsheet downloaded from http://openscienceframework.org/project/ixGcd/.

## Results

### Pretest results

A direct comparison between pre-test and post-test adaptation effect was not performed because of the different conditions under which each SJ test was taken (no action vs. action task, respectively). However, it is interesting to investigate pre-test values across the conditions to control for potential baseline differences in PSS or SD between the experimental groups assigned to different action types, previous to any adaptation. Previous studies have shown that greater individual PSS shifts are associated to participants with larger SD (broader SJ curves) in SJ tasks (Van der Burg et al., 2013). Two separate one-way ANOVAs were conducted on the individual PSS and SD values with action type (grouping vs. segregation vs. control) as a between participants factor. The results showed no statistically significant differences in PSS between groups [*F*_(2, 45)_ = 0.720, *p* = 0.492, $\eta_{p}^{2}$ = 0.03], and only a marginal trend for an effect in SD [*F*_(2, 45)_ = 2.834, *p* = 0.069, $\eta_{p}^{2}$ = 0.11]. To further investigate this trend, a Games-Howell *post-hoc* test [non-parametric was used because lack of homocedasticity; Levene's test *F*_(2, 45)_ = 3.197, *p* = 0.05] revealed that the SD in the grouping condition (98 ± 45 ms, values corresponding to the mean ± standard deviation) was larger than in the control condition (66 ± 21 ms, *p* = 0.044, Cohen's ds = 0.91, Hedges's g s = 0.89), but was not different from the segregation condition (87 ± 45 ms, *p* = 0.769, Cohen's ds = 0.24, Hedges's g s = 0.24). SD in the segregation condition was not different from the control condition either (*p* = 0.231, Cohen's ds = 0.60, Hedges's g s = 0.58). Thus, according to these results, and bearing in mind a possible relationship between PSS and SD, only the interpretation of a significant difference in adaptation magnitude between control and grouping conditions would be under dispute. Since these two conditions did not in fact differ in PSS shift, these results ensure that the comparison between grouping vs. segregation and segregation vs. control condition could not be biased by baseline differences between groups. Indeed, because no differences were found in the SD analysis between grouping and control condition after adaptation (see results below), we suspect the marginal difference found in the SD during the Pretest for the control group (vs. grouping group) cannot account for the numerically smaller recalibration magnitude.

### SJ task

The analysis of the three experimental conditions allowed comparing the consequences of the different action types performed during the adaptation period, on audio-visual recalibration. In particular, we examined the PSS shift between the two adaptation directions VA and AV, which is an estimate of the magnitude of recalibration. Hence, we will first report the recalibration results for adaptation under each action condition. Then, the comparison between the grouping and segregation conditions directly contrasted the effects of action type demands (promoting grouping or segregation of flash-tone pairs) on the magnitude of temporal recalibration. Here, the grouping condition was expected to lead to stronger recalibration, compared to the segregation condition. A third condition (type of action), was included to control for possible differences introduced by the larger number of actions in the segregation condition, compared to the grouping condition. The number of actions in the control condition was larger than in the segregation condition, whilst the strength of recalibration predicted by action-event synchrony was in principle expected to be equivalent to the grouping condition.

#### Recalibration magnitude as a function of action type during adaptation

**Figure 2:**
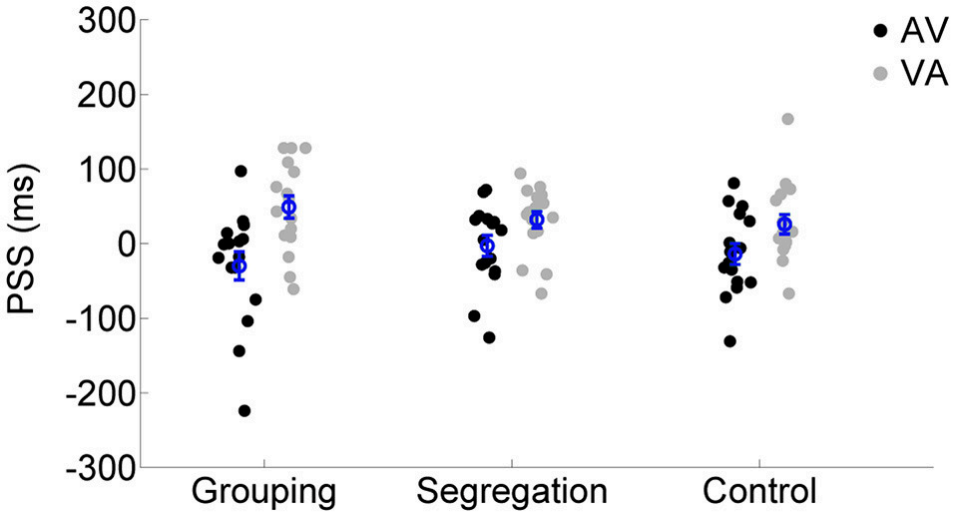
**Individual PSS values (ms) after flash lagging tone (AV) and flash leading tone (VA) adaptation phase for each action type condition (grouping, segregation, and control)**. The blue circles depict the mean PSS with the corresponding standard error of the mean.

**Figure 3:**
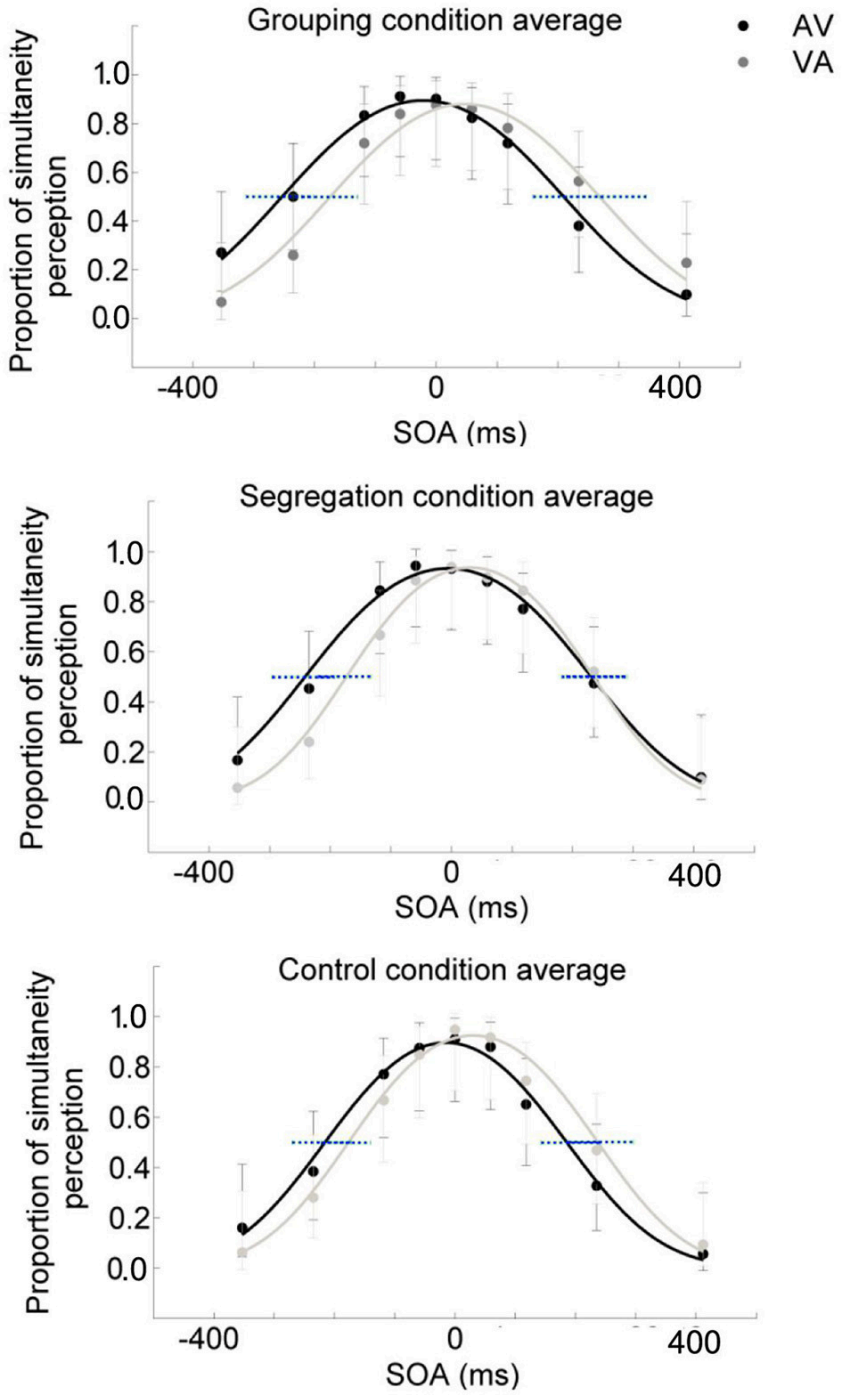
**Average psychometric function for the grouping (A)**, segregation **(B)** and control **(C)** action type groups after adaptation to flash lagging tone (AV) and flash leading tone (VA) conditions, in dark and light gray, respectively. Data points in dark and light gray for AV and VA, respectively. 95% confidence intervals for the A50V and V50A boundaries were depicted in blue dashed lines.

To assess whether there is an interaction between action type and adaptation magnitude, a mixed model ANOVA was conducted on PSS with the direction of adapted lag (AV vs. VA) as a within participants factor (indexing recalibration magnitude) and action type condition (grouping vs. segregation vs. control) as a between participants factor (see Figure 2). PSS values were obtained from the resulting psychometric functions after fitting the SJ data (average functions for each action type condition are shown in Figures 3A–C).

There was a main effect of the adapted lag [*F*_(1, 45)_ = 48.438, *p* < 0.001, $\eta_{p}^{2}$ = 0.52, $\eta_{G}^{2}$ = 0.17], revealing a general effect of recalibration in the expected direction and, more interestingly, an interaction between action type and lag [*F*_(2, 45)_ = 3.550, *p* = 0.037, $\eta_{p}^{2}$ = 0.14, $\eta_{G}^{2}$ = 0.03] that revealed possible differences in the recalibration effect across action type conditions. In order to investigate the significant interaction, we examined the recalibration after-effects within each action type condition separately, using a one tailed, *t*-test of related samples on the PSS values after adaptation to AV and VA as a within participants factor.

**Figure 4:**
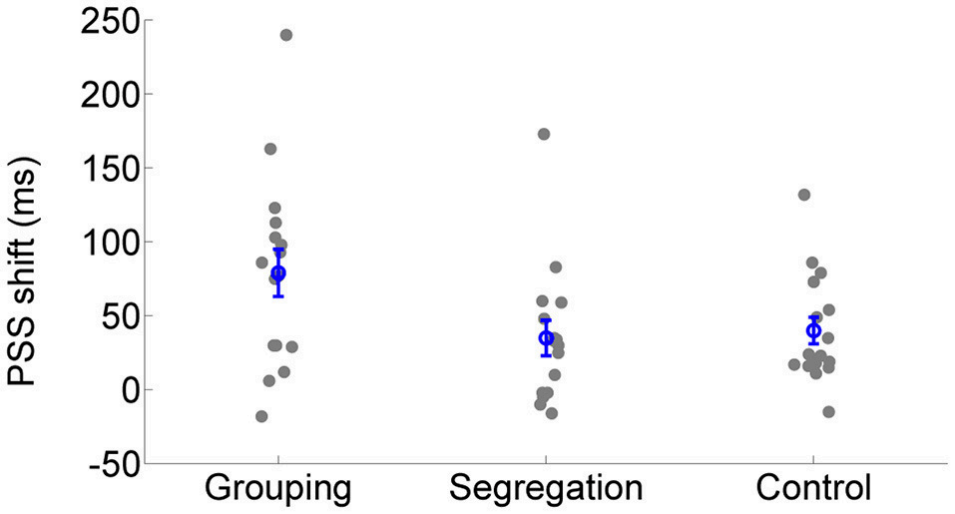
**Individual recalibration magnitude (PSS shift) in the grouping, segregation and control condition**. The blue circles depict the mean PSS shift with the corresponding standard error of the mean.

All action types produced significant recalibration after-effects (PSS shifts) in the predicted direction; grouping [average PSS shift = 79 ± 65 ms SD, *t*_(15)_ = 4.834, *p* < 0.001, Cohen's dz = 1.21, Hedges's g av = 1.12], segregation [PSS shift = 35 ± 47 ms, *t*_(15)_ = 3.006, *p* = 0.009, Cohen's dz = 0.75, Hedges's g av = 0.68] and control [PSS shift = 40 ± 37 ms, *t*_(15)_ = 4.288, *p* = 0.001, Cohen's dz = 1.072, Hedges's g av = 0.71]. In order to follow up possible differences in the size of recalibration, as indicated by the interaction, we conducted a one-way ANOVA with recalibration magnitude (PSS shift = PSS after adaptation to flash lagging-PSS after adaptation to flash leading). Recalibration magnitudes (PSS shifts) are depicted in Figure 4. The ANOVA showed that there is a significant effect in the PSS shift between action type groups [*F*_(2, 45)_ = 3.584, *p* = 0.036, $\eta_{p}^{2}$ = 0.14]. A Tukey *post-hoc* test revealed that the PSS shift was larger in the grouping condition (79 ± 65 ms, *p* = 0.047, Cohen's ds = 0.78, Hedges's g s = 0.76) than in the segregation condition (35 ± 47 ms). This is in line with our initial hypothesis and the prediction of larger recalibration in the grouping condition than in the segregation condition. However, the recalibration magnitude in the control condition was similar to the one obtained in the segregation condition (40 ± 37 ms; *p* = 0.957, Cohen's ds = 0.12, Hedges's g s = 0.12), and much smaller (a marginal trend) than in the grouping condition (*p* = 0.088, Cohen's ds = 0.74, Hedges's g s = 0.72). This was not in line with our prediction.

#### Temporal window of simultaneity (SD)

**Figure 5:**
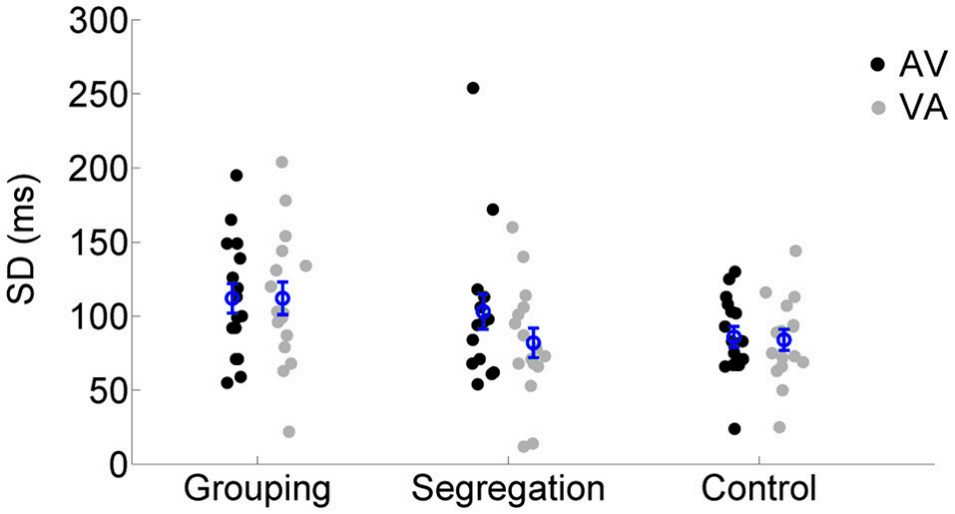
**Individual SD estimates for each adaptation condition (flash lagging tone-AV and flash leading tone-VA) in the grouping, segregation and control action type group**. The blue circles depict the mean SD with the corresponding standard error of the mean.

We also examined the SD values of the SJ curves (reflecting the temporal window of simultaneity) across the different action type conditions using a mixed model ANOVA. SD values for each adaptation condition and action type conditions are reported in Figure 5. None of the terms in the analysis resulted significant [neither the interaction, *F*_(2, 45)_ = 1.633, *p* = 0.207, $\eta_{p}^{2}$ = 0.07, η*G*^2^ = 0.02, nor the main effects of lag, *F*_(1, 45)_ = 2.444, *p* = 0.125, $\eta_{p}^{2}$ = 0.05, $\eta_{G}^{2}$ = 0.01 or action type *F*_(2, 45)_ = 2.564, *p* = 0.088, $\eta_{p}^{2}$ = 0.10, $\eta_{G}^{2}$ = 0.08].

### Synchronization task

**Figure 6:**
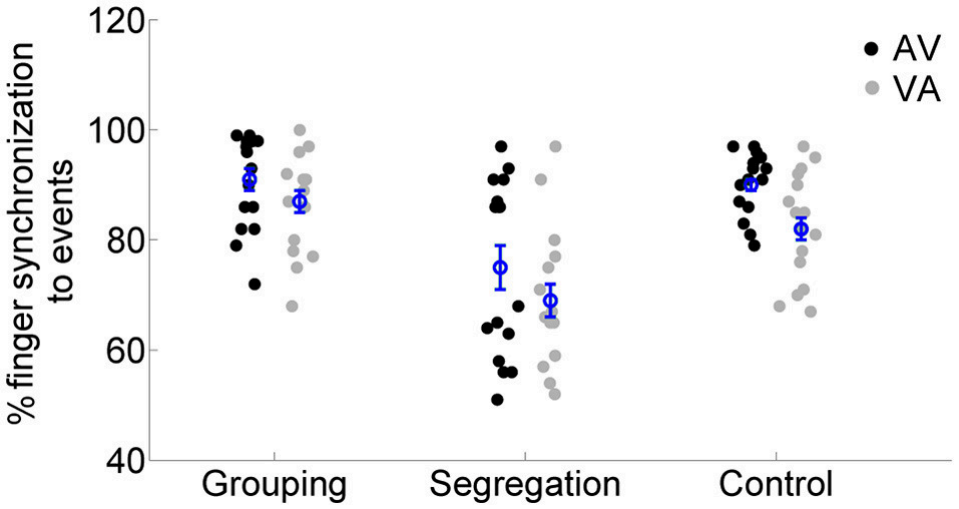
**Individual percentage of synchronization performance for each action type (grouping, segregation and control groups) after flash lagging tone (AV) and flash leading tone (VA) adaptation conditions**. The blue circles depict the mean synchronization with the corresponding standard error of the mean.

Finally, we examined the response time performance in the motor synchronization task, which participants were asked to perform during adaptation. We run a mixed model ANOVA on synchronization performance values (see analysis section) with the factors action type and adaptation lag. The results (see Figure 6) showed no interaction between action type and adaptation lag [*F*_(2, 45)_ = 0.385, *p* = 0.683, $\eta_{p}^{2}$ = 0.02, $\eta_{G}^{2}$ = 0.01], but the main effects of adaptation lag [*F*_(1, 45)_ = 10.098, *p* = 0.003, $\eta_{p}^{2}$ = 0.18, $\eta_{G}^{2}$ = 0.07] and of action type [*F*_(2, 45)_ = 17.546, *p* < 0.001, $\eta_{p}^{2}$ = 0.44, $\eta_{G}^{2}$ = 0.36] reached significance. A *post-hoc* analysis [we applied Games Howell, due to unequal variances between action types in the AV condition, Levene's test, *F*_(2, 45)_ = 27.225, *p* < 0.001] revealed that participants in the segregation group (75 ± 16% and 69 ± 12% for AV and VA, respectively) had overall lower synchronization performance compared to the grouping (91 ± 9% and 87 ± 9%, *p* < 0.01, Cohen's ds = 1.06, Hedges's g s = 1.03) and control group (90 ± 6% and 82 ± 10%, *p* < 0.01, Cohen's ds = 1.19, Hedges's g s = 1.16). But no differences in synchronization performance were found between the grouping and control group (*p* = 0.594, Cohen's ds = 0.07, Hedges's g s = 0.07).

## Discussion

The human perceptual system is sensitive to temporal differences in the order of 10 microseconds, for example to capitalize on inter-aural time differences to locate sound sources (Leshowitz, 1971), or voicing onset time differences for phonological discrimination in speech perception (Lisker and Abramson, 1964). However, across sensory modalities, the estimation of event order is much less reliable due to the various sources of asynchrony, including varying processing times across sensory pathways (e.g., King, 2005). One consequence is that cross-modal simultaneity is often experienced over a relatively large temporal window, in the order of tenths of a second, called the “window of simultaneity” (e.g., Meredith et al., 1987) whose width is flexible to a certain extent (Powers et al., 2009; Stevenson et al., 2014). The various sources of temporal uncertainty make us wonder about the particular role of physical synchrony in multisensory perception. We speculate that physical synchrony, alone, is not decisive to understand which information is ultimately grouped or segregated in perception. Instead, other processes such as previous experience within the current context and direct interaction with the environment might help building up multisensory percepts via structuring the temporal organization of events. Along with other studies (Parsons et al., 2013; Desantis et al., 2016) we hypothesize that systematic interactions between actions and sensory events of different modalities might lead to particular temporal adjustments in the perception of cross-modal simultaneity, or temporal order.

The aim of this study was to examine whether actions can help structure temporal organization of sensory events in different modalities (auditory and visual) via their temporal relationship with the observer's actions. Our logic capitalized on the varying patterns of synchronization between a motor task and cross-modal sensory events used for adaptation, in a recalibration paradigm. We assumed that certain action synchronization patterns would promote cross-modal grouping whereas others would induce segregation of otherwise identical audio-visual events. We predicted larger recalibration magnitudes when actions during the adaptation phase putatively promoted grouping (grouping and control condition) rather than segregation (segregation condition) of sensory events across modalities.

The results showed that recalibration after-effects happened for all the conditions (grouping, segregation and control), attesting to the strength of the recalibration paradigm. Interestingly, the recalibration effect (measured as PSS shift between opposite adaptation directions) was more than twice as large in the grouping condition than in the segregation condition, supporting our initial prediction. However, one difference between the grouping and segregation conditions was that the synchronization task during adaptation required more demanding, quicker action synchronization to sensory events. For this reason, the design also included a control condition meant to reproduce grouping, but with task difficulty commensurate to, if not higher than, the segregation condition. The size of recalibration in this control condition tended to be smaller than in the grouping condition (39 ms smaller PSS shift; marginal trend), and, not different from the PSS shift measured in the segregation condition. For this reason, our interpretation about the effects (or the nature) of actions on sensory recalibration in this paradigm must be cautious.

On the one hand, the grouping condition induced the largest recalibration after-effects (79 ± 65 ms) and, on the other hand, the segregation and control conditions, led to lesser, albeit significant, after-effects (35 ± 47 ms and 40 ± 37 ms, respectively). One possibility is that the systematic relation between actions and sensory events that we considered to produce grouping (in the case of the grouping and control condition) or segregation (in the segregation condition) of events, might not have been the only factor accounting for the diminished recalibration magnitude in the control and segregation condition. Instead, other factors, such as the level of motor difficulty could have influenced the size of recalibration. Though difficulty was not measured directly, success in the synchronization task provides an indirect index of synchronization performance. Based on this index for the synchronization task, it is worth noting that synchronization performance was equivalent in the grouping and control conditions, which ended up leading to different audio-visual recalibration magnitudes. According to this, the decrease in recalibration magnitude of the segregation and control condition could not be explained only by a difference in motor task difficulty.

Since motor task difficulty during adaptation might not fully account for the modulation in the size of recalibration, we entertain two further possible interpretations regarding the reduced recalibration magnitude in the control condition. One option is that number of actions required for each cross-modal pair in the adaptation would be proportional to cognitive load which, in turn, determined the size of the recalibration magnitude. Along these lines, we speculate that attention was deployed to the preparation and execution of synchronized actions and hence, diverted away from the sensory events, producing a weaker adaptation and recalibration. This interpretation of results can explain the decreased recalibration magnitude in the control and segregation conditions, and is in line with the idea that diverting attention away from the sensory events, or their temporal relation, modulates temporal recalibration (Heron et al., 2010; Ikumi and Soto-Faraco, 2014). A second possible account to be considered for the present pattern of results would be that the motor task in the control condition was not effective in promoting the intended grouping of cross-modal events. This possibility is difficult to rule out, since there is no objective measure of grouping. In the control condition, participants were asked to synchronize an action with the first sensory event of the pair, and to perform two extra finger actions outside the interval of presentation of the flash-beep pair. However, in hindsight, this organization might have induced participants to anticipate the first event of the pair, and then quickly switch attention to the offset of the second event, a pattern very similar to what the segregation condition intended to accomplish. Hence, there is a chance that the control condition might have, in the end, promoted segregation and in consequence weaker grouping of audio-visual events. It is not possible to discard this account, and therefore the interpretation of the present results must remain undecided, about this particular aspect.

In fact, the two possible accounts of the present pattern of results discussed above are not exclusive of each other, and both involve non-trivial ways in which attention does affect the potential of cross-modal events for recalibration. On the basis of these considerations, we propose one encompassing explanation: the conditions requiring more actions per cycle in the course of adaptation (segregation and control tasks) might have diverted the focus of attention from the sensory events altogether, and/or created separate attention episodes for each sensory event. In both cases, the focus of attention would have led to the consequence of reducing the magnitude of the perceptual temporal adjustment. The reduction of cross-modal recalibration by diverting the focus of attention away from the stimuli or temporal relation between events is in line with Heron et al. (2010). Also, modulation of recalibration caused by placing attention to one or another particular event of the cross-modal pair have been demonstrated before (Ikumi and Soto-Faraco, 2014). Further, we advance the tentative hypothesis that in this case, attention to actions during adaptation in our temporal recalibration paradigm, might have recruited similar anticipatory processes required to calibrate perceptual events. This hypothesis would be in line with the proposal that some core predictive mechanisms may be shared by sensory and motor systems (Schubotz, 2007; Engel and Fries, 2010). We speculate that competition for the predictive mechanisms by means of attention might have reduced the effectiveness of temporal recalibration for cross-modal events during the perceptual process.

In conclusion, given the remarkable variability in arrival timings within and across sensory modalities in multisensory environments, the presence of reliable temporal anchors might play a fundamental role helping resolve which cross-modal events would be grouped or segregated in time. We hypothesized that actions might supply such reliable temporal anchors. Our results, although not completely conclusive in this respect, nevertheless suggest an indirect route whereby the allocation of attention to the planning/execution of concurrent actions can indeed modulate temporal recalibration of audio-visual events. We believe that these results add to the previous literature showing that participants' inner state, including motor and cognitive factors plays an important role in synchronization of cross-modal sensory events (Heron et al., 2010; Parsons et al., 2013; Ikumi and Soto-Faraco, 2014). The new insight is that the role of attention modulations in the re-alignment of multisensory events might, in fact, be as a mediator for the impact of the motor system in temporal perception.

## Author contributions

Study conception and design: SS, NI. Acquisition of data: NI. Analysis and interpretation of data: SS, NI. Drafting of manuscript: SS, NI.

## Funding

This research was supported by the Spanish Ministry of Science and Innovation (PSI2016-75558-P), Comissionat per a Universitats i Recerca del DIUE- Generalitat de Catalunya (2014SGR856), and the European Research Council (StG-2010 263145). The funders had no role in study design, data collection and analysis, decision to publish, or preparation of the manuscript.

### Conflict of interest statement

The authors declare that the research was conducted in the absence of any commercial or financial relationships that could be construed as a potential conflict of interest.

## Footnotes

^1^The tapping synchronization task is interesting as it involves the alignment of actions to external sensory events and it is characterized by an anticipation of the action, as if the sensory events were taken as a consequence of the action (Yarrow and Obhi, 2014).

^2^Despite we initially used a fourth parameter for the estimation of different sources of noise (see Yarrow et al., 2011) during the piloting phase of the project, we realized that the bootstrapping procedure for the estimated confidence intervals took much time to converge (up to 40 minutes) and did not improve significantly the fits of the first participants (differences of deviance fit values were not larger than the deviance improvement predicted with the addition of 1 parameter. *X*^2^(1 df) = 3.84). Thus, we opted for the original three parameter model.
