# Supplementary material for: Grouping and Segregation of Sensory Events by Actions in Temporal Audio-Visual Recalibration
Source: Front Integr Neurosci. 2017 Jan 19;10:44. doi: 10.3389/fnint.2016.00044 (PMC5243829; doi:10.3389/fnint.2016.00044)
Supplement: Supplementary file 1 [file DataSheet1.docx]

# Pilot Study (sensory-sensory recalibration)

It consisted of a classic temporal recalibration paradigm on audio-visual pairs, without any motor task performed during adaptation. To control participants focus attention to the events during the adaptation; they performed an odd-ball detection task on deviant stimuli; either a tone 16% lower in frequency (1.5 kHz, ~60 [A]dB SPL) or a visual stimulus 35% smaller (outer and inner diameter, 2.75° and 1.38°, respectively). Detection responses were considered correct when falling within 650 ms after odd-ball presentation. Deviants appeared with a probability of 2.5% during the adaptation and re-adaptation trials. If the deviant was not detected, the following test trial was removed from the analysis, and repeated at the end of the block.

The analysis of the preliminary pilot study (N = 6), in which no motor task was performed, was conducted through a Wilcoxon signed rank test of related samples. The results indicate that flash-tone pairs at a lag of 470 ms led to large recalibration (PSS shift = 83 ± 36 ms), that is the distance between PSS values after exposure to flash-leading-tone pairs and flash-lagging-tone pairs (Z = -2.201, p = 0.028). The results of the pilot confirm that our experimental procedure promotes recalibration of perceptual events when no motor task is involved during the adaptation. The estimated SD, A50V and V50A boundaries are reported with the averaged 95% CI in the Table 1.

Table 1: A50V, V50A, SD and CI mean estimates for each adaptation condition (flash lagging tone-AV and flash leading tone-VA) in the pilot study.

|  | A50V | V50A | SD | CI A50V | CI V50A | CI SD |
| --- | --- | --- | --- | --- | --- | --- |
| AV | -279 ms | 174 ms | 83 ms | [-358 ms, -235 ms] | [130 ms, 229 ms] | [53 ms, 135 ms] |
| VA | -160 ms | 223 ms | 81 ms | [-203 ms, -125 ms] | [173 ms, 291 ms] | [54 ms, 139 ms] |

# Boundaries (A50V and V50A estimates) of the SJ task

As mentioned in the analysis section, the boundaries (A50V and V50A) for each adaptation to AV and VA conditions are only analyzed for completeness, lacking of any prior hypothesis. A 3 way mixed ANOVA was analyzed with action type as a between subjects factor and boundaries and lag as a within participants factor. The three way interaction was not significant (F_(2,45)_ = 1.85, p = 0.169) and thus, the combined effect of the boundaries and their simultaneity perception values after adaptation to AV or VA pairs is the same for grouping, segregation and control conditions. As expected, there were significant main effects of boundaries (F_(1,45)_ = 677.72, p < 0.01) and of lag (F_(1,45)_ = 48.31, p < 0.01) but not a main effect of action type (F_(2,45)_ = 0.09, p = 0.913). Furthermore, there was a significant interaction between the simultaneity perception after lag adaptation and the action type performed during adaptation (F_(2,45)_ = 3.55, p = 0.037), but not significant interaction was found between boundaries and action type (F_(2,45)_ = 0.792, p = 0.459) or between boundaries and lag (F_(1,45)_ = 3.075, p = 0.086). Overall, these results suggest the boundaries of the SJ curve (audition leading and vision leading side) were equally affected in the grouping, segregation and control conditions after adaptation to AV and VA. The A50V and V50A values and the corresponding 95% CI averages for each adapted condition for the action type groups were described in the Table 2.

Table 2: A50V, V50A and CI mean estimates for each adaptation condition (flash lagging tone-AV and flash leading tone-VA) in the grouping, segregation and control action type group.

|  | A50V | | V50A | | CI A50V | | CI V50A | |
| --- | --- | --- | --- | --- | --- | --- | --- | --- |
|  | AV | VA | AV | VA | AV | VA | AV | VA |
| Grouping | -262 ms | -174 ms | 203 ms | 272 ms | [-337 ms, -205 ms] | [-228 ms, -129 ms] | [143 ms, 256 ms] | [199 ms, 343 ms] |
| Segregation | -236 ms | -172 ms | 230 ms | 236 ms | [-297 ms, 187 ms] | [-215 ms, -136 ms] | [175 ms, 302 ms] | [190 ms, 307 ms] |
| Control | -215 ms | -178 ms | 188 ms | 230 ms | [-268 ms, -170 ms] | [-221 ms, -135 ms] | [146 ms, 243 ms] | [182 ms, 285 ms] |
